# How Twitter data sampling biases U.S. voter behavior characterizations

**DOI:** 10.7717/peerj-cs.1025

**Published:** 2022-07-01

**Authors:** Kai-Cheng Yang, Pik-Mai Hui, Filippo Menczer

**Affiliations:** Observatory on Social Media, Indiana University, Bloomington, Indiana, United States

**Keywords:** Twitter, Election, Voter, Bias, Data sampling

## Abstract

Online social media are key platforms for the public to discuss political issues. As a result, researchers have used data from these platforms to analyze public opinions and forecast election results. The literature has shown that due to inauthentic actors such as malicious social bots and trolls, not every message is a genuine expression from a legitimate user. However, the prevalence of inauthentic activities in social data streams is still unclear, making it difficult to gauge biases of analyses based on such data. In this article, we aim to close this gap using Twitter data from the 2018 U.S. midterm elections. We propose an efficient and low-cost method to identify voters on Twitter and systematically compare their behaviors with different random samples of accounts. We find that some accounts flood the public data stream with political content, drowning the voice of the majority of voters. As a result, these hyperactive accounts are over-represented in volume samples. Hyperactive accounts are more likely to exhibit various suspicious behaviors and to share low-credibility information compared to likely voters. Our work provides insights into biased voter characterizations when using social media data to analyze political issues.

## Introduction

In recent years, social media have served as important platforms for news dissemination and public discussion of politics. With more citizens consuming information and actively expressing their political opinions online, political figures, governments, and agencies have started to adopt social media to reach out to the public and amplify their influence (Jungherr, 2016). These activities generate huge amounts of valuable data, making it possible to study voter behavior (Small, 2011; Jungherr, 2014; Ausserhofer & Maireder, 2013) and estimate public opinions around political issues (Diakopoulos & Shamma, 2010; Wang et al., 2012) through computational approaches. Some researchers even attempt to predict election outcomes (Tumasjan et al., 2010; DiGrazia et al., 2013; Burnap et al., 2016), although the reliability of such predictions has been questioned (Gayo-Avello, Metaxas & Mustafaraj, 2011).

**Figure 1:**
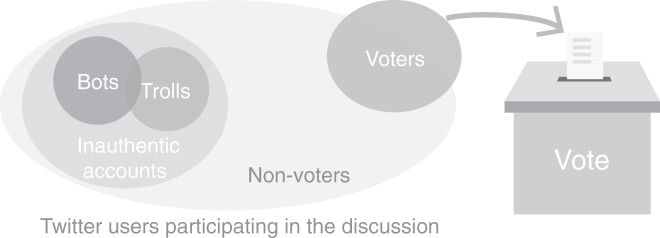
The composition of different sets of accounts participating in election-related discussion on Twitter. The voters determine real-world outcomes, while the online data comes from a population that includes non-voters and inauthentic accounts. The sizes of the sets in the diagram do not correspond to the actual numbers of accounts in each group.

However, raw social media data might be seriously biased and even lead to false signals. To demonstrate, we use Twitter as an example of social media platforms given its popularity among U.S. politically active citizens. Figure 1 illustrates the composition of different types of accounts that participate in the online discussion about elections. Ideally, we want to focus on the data generated by real-world voters since they determine the outcomes of elections. But the data stream originates from a population that does not contain all voters and that contains non-voters and inauthentic accounts. The latter group includes entities like malicious social bots, foreign trolls that have been shown to manipulate public opinion regarding elections, and more (Bessi & Ferrara, 2016; Deb et al., 2019; Stella, Ferrara & De Domenico, 2018; Ferrara, 2017; Badawy, Ferrara & Lerman, 2018; Shao et al., 2018; Zannettou et al., 2019a). Relying on content generated by such accounts is clearly problematic.

To understand potentially biased characterizations of U.S. voters based on observations of Twitter data, we propose a method to extract self-identified voters during the 2018 U.S. midterm elections and use the resulting group as a baseline. We also propose two variants of this methods that are more efficient to implement. We further create two different samples of accounts. First, naive sampling based on tweet volume leads to over-representation of hyperactive accounts. Second, random resampling of unique accounts removes this bias. In this exploratory analysis, we systematically compared the accounts selected according to these four different methods to the baseline. We examined account characteristics such as their profiles, activity in the discussion, general sentiment expression, and political alignment. To quantify the prevalence of different questionable behaviors, we also analyzed their sharing of low-credibility information, signs of automation, and so on.

We found that hyperactive accounts are more likely to exhibit suspicious behaviors and share low-credibility information compared to likely voters. These findings further our understanding of biases in characterizations of U.S. voters based on Twitter data, providing useful guidance toward political studies on social media. For journalists and the public at large, it reveals important insights about the accounts they encounter online everyday.

## Related Work

### Politics-related social media studies

Many recent computational social science and political science studies are dedicated to understanding online behaviors of voters in political discussions. To name a few, Small (2011) studies the usage of political hashtags. Jungherr (2014) investigates the logic of political coverage on Twitter by comparing it with traditional media. And Ausserhofer & Maireder (2013) study interrelations between the users.

Another line of work seeks to predict election outcomes using indicators estimated from social media data. In the context of a German election, Tumasjan et al. (2010) show that the number of tweets can reflect the election result. A study by DiGrazia et al. (2013) similarly finds a correlation between the votes and candidate name mentions on Twitter in the 2010 U.S. midterm elections while accounting for other predictors. Considering the same elections, the model proposed by Livne et al. (2011) uses variables derived from Twitter content and network features and achieves decent prediction performance. Other studies depend on the sentiment of the tweets. For example, O’Connor et al. (2010) found that tweet sentiment is correlated with presidential job approval and consumer confidence polls. Ceron et al. (2014) demonstrate the usefulness of sentiment analysis in multiple elections. Burnap et al. (2016) show similar results in the 2015 U.K. general election. Despite these efforts, the reliability of electoral predictions based on social media data has been questioned (Gayo-Avello, Metaxas & Mustafaraj, 2011).

### Questionable actors and behaviors

The politics-related social media studies reviewed above usually assume that social media data comes from real users and barely mention inauthentic accounts. This is not surprising, as studies of inauthentic actors on social media have only started to emerge in recent years. Here we briefly cover the studies about malicious social bots and trolls.

Social bots are social media accounts controlled completely or in part by algorithms (Stocking & Sumida, 2018; Ferrara et al., 2016). Their automated nature allows a bot creator to easily generate large amounts of content or create false appearance of popularity. Reports show that malicious bots were involved in recent elections in Europe and the U.S. (Bessi & Ferrara, 2016; Deb et al., 2019; Stella, Ferrara & De Domenico, 2018; Ferrara et al., 2020; Gorodnichenko, Pham & Talavera, 2021). Close analyses suggest that bots tend to have suspicious profile information, recent creation dates, large numbers of tweets, and limited numbers of original posts (Ferrara et al., 2016). Questionable behaviors by malicious bots include astroturfing (Conover et al., 2011), spreading misinformation (Shao et al., 2018), and using inflammatory language to irritate others (Stella, Ferrara & De Domenico, 2018).

In this article we use the term “trolls” to refer to fake accounts controlled by state-sponsored agencies. Studies suggest that these accounts have interfered with the 2016 U.S. presidential election (Badawy, Ferrara & Lerman, 2018; Zannettou et al., 2019b, 2019a). The characteristics of the trolls can be different from those of random Twitter accounts: they tend to use deceptive language (Addawood et al., 2019), their posts come from different Twitter clients, and their creation dates are concentrated (Zannettou et al., 2019a).

Note that the bot and troll behaviors mentioned above may only represent a subset of inauthentic behaviors online; other types of suspicious behaviors may remain unnoticed. As a result, our understanding of the prevalence of questionable behaviors among typical Twitter users is limited.

### Voter identification

On social media, voters are often identified by conducting surveys. Mellon & Prosser (2017) used a nationally representative sample in the U.K. and find that Twitter and Facebook users tend to be younger, better-educated, more liberal, and to pay more attention to politics compared to voters at large. The Pew Research Center publishes a series of studies in which they first survey a group of U.S. adults from a nationally representative panel for their demographics and political ideology. Then they link the survey data to Twitter handles shared by the participants (Wojcik & Hughes, 2019; Hughes et al., 2019). Among other things, they find that a small portion of users generate most of the political content on Twitter and that politics is not the main topic of conversation for voters.

Another approach starts from social media handles and then attempts to establish links to voters. Barberá (2016) proposed a method to match geolocated tweets with voting registration records to obtain the demographics of the people behind the Twitter handles. Grinberg et al. (2019) matched Twitter profiles against voter registration information, finding that a small portion of U.S. voters account for the majority of fake news exposure and sharing during the 2016 presidential election.

The approaches introduced above can be powerful in connecting online behaviors and demographics of voters. However, high quality data sources like nationally representative panels, voting registration records, and large-scale surveys are expensive and out of reach for many researchers. Moreover, these approaches can hardly be automated. In their recent work, Deb et al. (2019) collected tweets related to the 2018 U.S. midterm elections and treated accounts tweeting the hashtag *#ivoted* on election day as actual voters. This method can be applied to large-scale data efficiently and thus makes rapid or even real-time analysis possible, although we show here that it introduces some bias.

## Data

### Tweet collection

In this study, we focus on Twitter data around the 2018 U.S. midterm elections. To create a dataset containing most of the election-related content, we collect tweets that include any hashtag in a query through Twitter’s free filtering API. The query includes a list of 143 election-related hashtags from a previous study (Yang, Hui & Menczer, 2019a). The list is initialized with several well-known hashtags like *#maga*, *#bluewave*, and *#2018midterms*. A snowball sampling method based on co-occurrence is then applied to expand the list (Conover et al., 2011). Hashtags used for each state’s Senate election (*e.g*., *#AZsen* and *#TXsen*) are also added to the list. We then manually check the list and remove irrelevant hashtags. This method is designed to capture most of the election-related tweets with high precision; although the list was not updated during data collection, newly emerging political hashtags likely co-occurred with those already on the list. The collection started on 05 October, 2018 and continued until the end of 2018, resulting in a dataset with about 60 M tweets by 3.5 M unique users.

### Naive accounts sampling

Our goal is to understand the characteristics of typical Twitter users and compare them with identified voters. For a meaningful comparison, we used the collected dataset as the sampling pool, *i.e*., we focus on accounts who discussed the 2018 U.S. midterm elections. We deployed two different methods to sample typical accounts. With an unweighted sampling method, we simply select unique accounts in our dataset randomly, hoping to get a representative sample. We call the group of accounts obtained with this sampling method ByAccount.

Sampling users from the tweets in our dataset favors active accounts: the chance of being selected is proportional to the number of tweets each account has in our dataset. We call the group of accounts sampled by this method ByTweet. In the following analyses, we will show that ByTweet accounts are hyperactive and generate disproportionately more content. As a result, Twitter users are more likely to be influenced by these accounts directly or indirectly, and content analysis based on the Twitter stream could be biased accordingly. The ByAccount and ByTweet groups have the same size as the Voter group, described next.

## Identification of Voters

In this section we present high-precision methods to identify likely voters on Twitter using the data collected. These accounts are used as a reference group to describe authentic behaviors in the online discussion regarding U.S. politics.

The basic idea is to use the messages and pictures posted by users on election day. We followed the approach of Deb et al. (2019) and used the hashtag *#ivoted* as a first filter. The hashtag was promoted by various social media platforms to encourage people to vote in the midterm elections, and it became quite popular for users to tweet about it after voting on election day (06 November, 2018). However, considering all accounts tweeting the *#ivoted* hashtag as voters may result in false positives since inauthentic accounts can also post the hashtag. In fact, Deb et al. (2019) exclude about 20% of the accounts that tweeted *#ivoted* in their analysis, based on high Botometer scores (Yang et al., 2019b).

**Figure 2:**
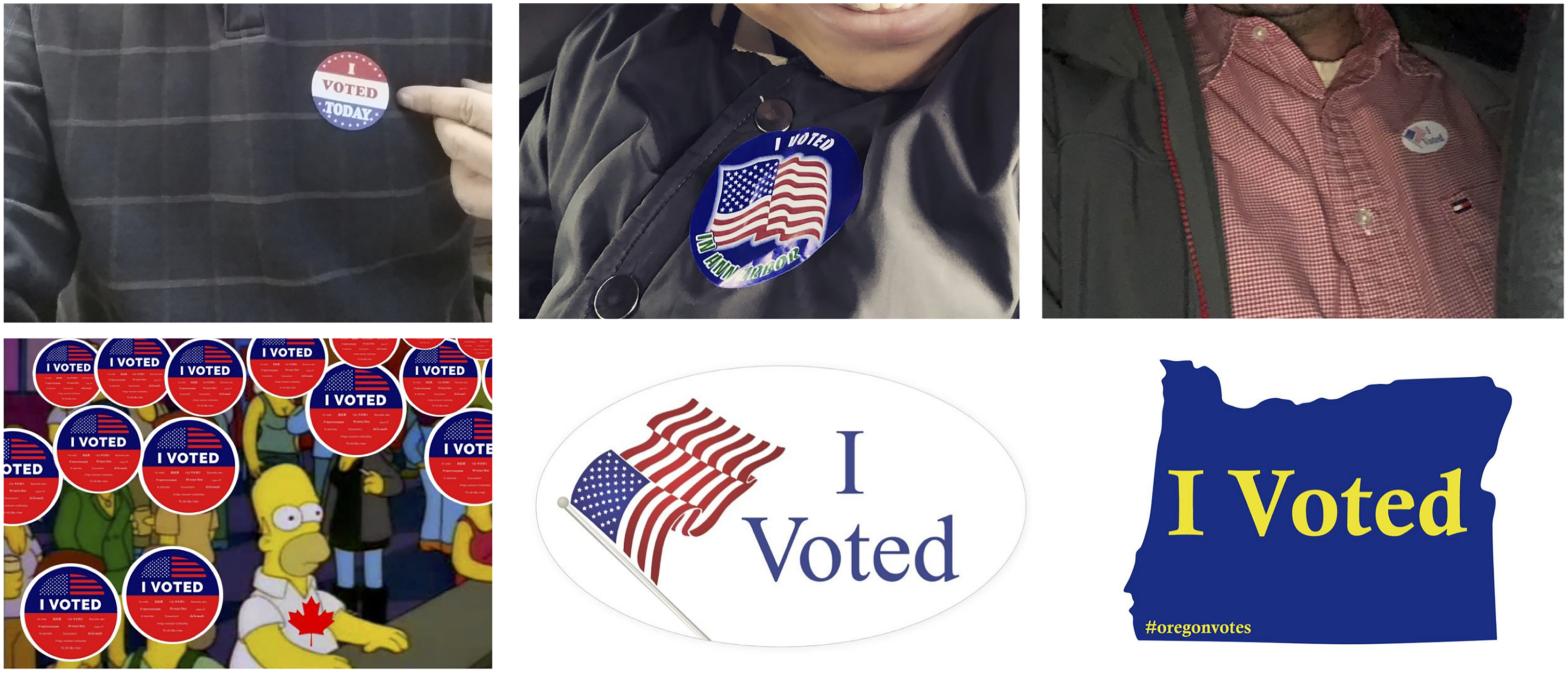
Examples of images providing positive (top) and negative (bottom) cases in our manual annotation of voters. Images are cropped for privacy consideration.

Instead of attempting to exclude all problematic accounts from those tweeting *#ivoted*, which is itself prone to false positive and false negative errors, we aimed to include only those who are very likely to be real voters. This approach yields voter accounts with high precision at the expense of recall, but this is acceptable for our purpose of characterizing likely voters. We notice that many voters shared pictures of themselves wearing “I Voted” stickers or buttons after voting (see Fig. 2). Based on this observation, we apply a second filter that verifies whether the *#ivoted* hashtag is accompanied by a picture showing a real person having voted.

### Manual annotation

To identify the voters in our dataset, we first extract all the tweets posted on election day that include both the *#ivoted* hashtag and images. We download all the pictures for manual annotation. A single criterion is used in the annotation: whether we can reach the conclusion that a real person just voted based on the picture. The annotations based on this criterion turn out to be quite straightforward. In most of the positive cases, the picture clearly shows a person with an “I Voted” sticker or button on their clothes or body. There are also some borderline cases where the pictures show voting-related memes or computer-generated “I Voted” stickers. Although the corresponding users might have voted, we still consider these as negative cases because sharing these pictures can be faked easily. Figure 2 shows examples of both positive and negative cases.

Although *#ivoted* is not on the list of hashtags used for collecting the tweets, it often co-occurs with other hashtags in the query, so we end up with 40,078 tweets containing this hashtag. Among these, 13,084 have pictures attached. Through annotation and data cleaning, we identify 8,092 voters. We call this group of accounts Voter. For comparison, we also sample an equal number of accounts that tweeted *#ivoted* with no pictures and call this group Ivoted.

The free Twitter API is sufficient for collecting the data. Manual annotation is relatively time-consuming: it took about 30 h for the first author to go through all the images. But the task is relatively straightforward, so no special training is needed. Moreover, implementing this method only requires basic programming skills and a Twitter developer account.

### Automated annotation

Since the human annotation is the bottleneck of the method described above, we also implemented an automated annotation process. The key challenge is to translate the annotation criterion into a format that machines can understand. Since most of the positive cases have the text “vote” in the picture, we operationalize the annotation criterion as identifying whether the picture contains the string “vot” (to match “vote,” “voted,” and “voting”).

Traditional optical character recognition software (https://opensource.google/projects/tesseract) does not provide acceptable performance. Therefore we resort to an off-the-shelf deep learning model provided by the Google Cloud service (https://cloud.google.com/vision/docs/ocr). We send all images to the the text detection API endpoint to extract text strings and check whether “vot” is in the response.

In our experiment, the automated approach annotates 46.5% of the pictures as positive. By treating the manual annotations as ground truth, we measure the performance of the automated approach: it achieves 0.81 in precision and 0.59 in recall. Close inspection of misclassified data reveals that most false positive cases are just like the ones in the bottom row of Fig. 2, as expected. Typical false negative cases, on the other hand, are mostly due to limitations of the API in handling blurred, distorted, and rotated pictures. For example, the API fails to detect the text in the upper right image in Fig. 2. We collect the accounts labeled as voters by the automated method and call the group Voter_auto.

With the help of Google Cloud or similar services, the whole voter identification process can be automated. The method is therefore fast and cheap; in our experiment, it takes less than 15 min and about $20 to annotate all the pictures. In summary, it is possible to setup an infrastructure to automatically identify likely voters on Twitter in real time at a very low price, if low recall is acceptable. With the rapid development of deep learning technologies, we expect to see an increase in performance and a decrease in price over time.

Table 1 summarizes the size of and methodology used to generate each group: Voter, Voter_auto, Ivoted, ByTweet, and ByAccount. As mentioned earlier, Voter contains 8,092 likely voters and we sample the same amount of accounts in Ivoted, ByTweet, and ByAccount while ensuring that they are mutually exclusive. Voter_auto and Voter share 4,852 accounts since they are generated from the same pool of accounts tweeting both *#ivoted* and images.

**Table 1:** Summary of the account groups examined in this study.

Account group	Size	Methodology
Voter	8,092	Manually-annotated likely voters from accounts tweeting *#ivoted* and photos
Voter_auto	5,920	Machine-identified likely voters from accounts tweeting *#ivoted* and photos
Ivoted	8,092	Accounts sampled randomly among those tweeting *#ivoted* but no photos
ByTweet	8,092	Accounts sampled based on tweet volume in the dataset
ByAccount	8,092	Accounts sampled randomly among all those in the dataset

We share the tweet IDs of the collected tweets and the user IDs of the users in different samples in a public data repository (https://doi.org/10.5281/zenodo.5866751). Sharing the raw tweets is prohibited by Twitter’s policy. But readers can use the IDs in our dataset to re-hydrate the data and reproduce the analysis.

## Characterization of Account Behaviors

In this section, we compare various characteristics of accounts in each group listed in Table 1. Our analysis covers account profiles, tweeting behaviors during the elections, sentiment expressions, and political alignment.

### Account profiles

**Figure 3:**
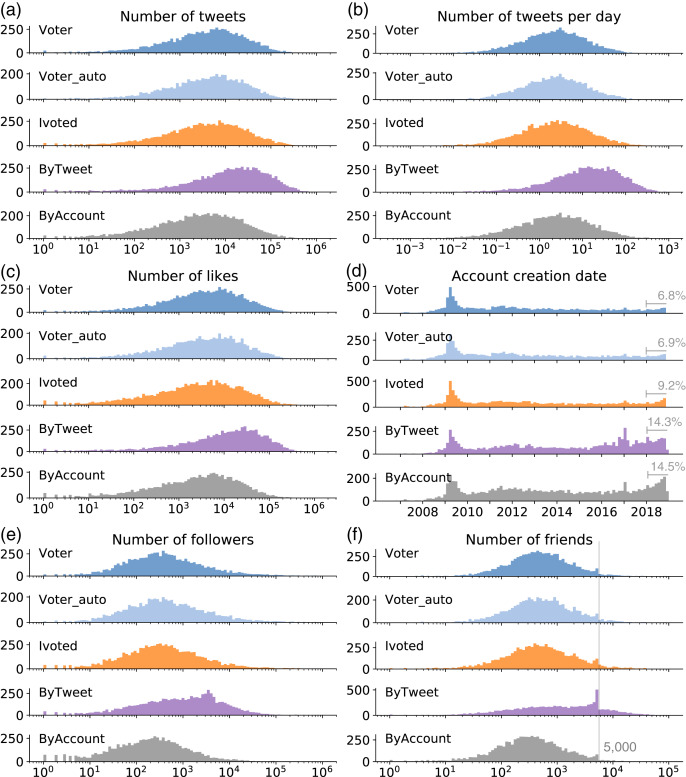
Distributions of (A) number of tweets; (B) number of tweets per day; (C) number of likes; (D) account creation date; (E) number of followers; and (F) number of friends for different groups. The annotations in (D) represent the percentage of accounts that were created in 2018 for each group. The line in (F) indicates 5,000 friends.

We systematically compare the profiles of the accounts in different groups and show selected results in Fig. 3. The information can be accessed directly from the user object embedded in each tweet, which reflects the status of the account at the time the tweet was collected. Since these user objects may change over time, we use the earliest version of each account in the collection for our comparison.

We show the distributions of total number of tweets in Fig. 3A. It appears that ByTweet accounts generate more tweets than other groups across their life time. Since the total number of tweets depends on the account age, we also show the distributions of number of tweets per day in Fig. 3B. This is calculated by dividing an account’s total number of tweets by its age, which is defined as the time difference (number of days) between the date of the data collection and the account creation date. Again, we can see that ByTweet accounts tweet faster than other groups. Over a half of the ByTweet accounts post more than 10 tweets per day; some post more than 100 tweets per day. The distributions of the two measures for other account groups are similar.

We also examine the number of likes and plot the distributions in Fig. 3C. Again, ByTweet accounts have more likes than other groups, which have identical distributions. The distributions of the number of likes per day (not shown) suggest that ByTweet accounts produce likes faster as well.

The distributions of account creation time are presented in Fig. 3D. Across all groups, we observe a peak around 2009, which is likely caused by the fast growth of Twitter during that year. ByTweet and ByAccount groups have more accounts created in 2018—right before the midterm elections—than other years. We annotate the percentage for each group in the figure to confirm this observation.

Figures 3E and 3F show the distributions of the numbers of followers and friends, respectively. The number of friends indicates how many other accounts each account is following. We find that ByTweet accounts tend to have more followers and friends than other groups. Interestingly, ByTweet has more accounts with about 5,000 friends. This is due to an anti-abuse limitation of Twitter, which states that an account cannot follow more than 5,000 friends unless it has more than 5,000 followers (https://help.twitter.com/en/using-twitter/twitter-follow-limit). The pattern here suggests that accounts in ByTweet are eagerly expanding their friend lists, until hitting the limit. ByAccount accounts tend to have fewer followers and friends than the voter groups.

Through the analyses of the account profiles, we find that ByTweet accounts are not only hyperactive in terms of tweeting, as expected given the sampling method; they are also hyperactive in liking and building social networks, and they tend to be more recent.

### Tweeting behaviors during elections

**Figure 4:**
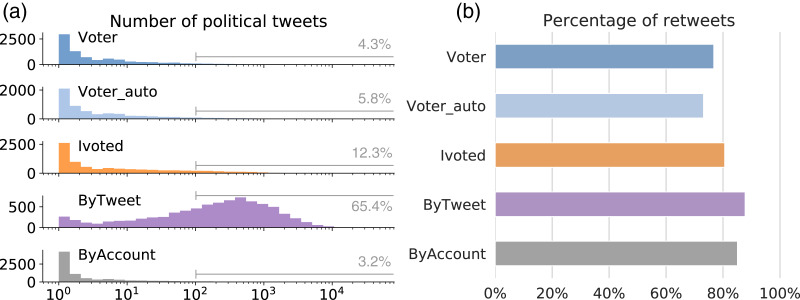
Tweeting behaviors during elections. (A) Distributions of the number of political tweets for different groups. Annotations show percentages of accounts with over 100 political tweets. (B) Percentage of retweets among the tweets generated by different groups.

We examined the level of participation in the online discussion about the midterm elections for different groups. This is measured by the number of tweets each account has in our dataset (see the distributions in Fig. 4A). The three voter groups have distributions similar to ByAccount, with accounts typically having fewer than a hundred political tweets. Due to the sampling method, it is expected that ByTweet accounts are more active in the discussion. The discrepancy in political activity is huge: over 65% of the ByTweet accounts have more than a hundred political tweets, compared to 12% or less in other groups.

These distributions suggest that the majority of the political tweets are generated by a small group of hyperactive users, which is in line with previous findings (Hughes et al., 2019). This is concerning when one considers that the volume of tweets is used to determine trending topics and viral content. Hyperactive accounts can flood the public data stream with content, drowning the voice of the majority of users, and manipulating engagement metrics and mechanisms that rely on these metrics, such as feed ranking and trending algorithms.

In addition to the absolute number of political tweets, we also examined the retweeting behaviors of different groups. On Twitter, one can amplify messages from other accounts by retweeting or quoting them. Quoting is relatively rare compared to retweeting, so we merge the two to simplify our analysis. For the political tweets generated by each group, we calculate the percentage of retweets and show the results in Fig. 4B. The majority of the tweets in each group are retweets, with ByTweet having a particularly high percentage.

### Sentiment analysis

Sentiment analysis is commonly adopted in mining social media for public opinions on political matters (Wang et al., 2012; Ramteke et al., 2016; Burnap et al., 2016). Here we aim to compare the sentiment expressions of different groups. We use VADER, an efficient lexicon- and rule-based sentiment analysis tool that works well on Twitter data (Hutto & Gilbert, 2014). In VADER’s lexicon dictionary, each word and symbol is assigned a sentiment score through crowdsourcing. When applied to a tweet, VADER estimates the negative, positive, and neutral expressions to generate an overall sentiment score.

**Figure 5:**
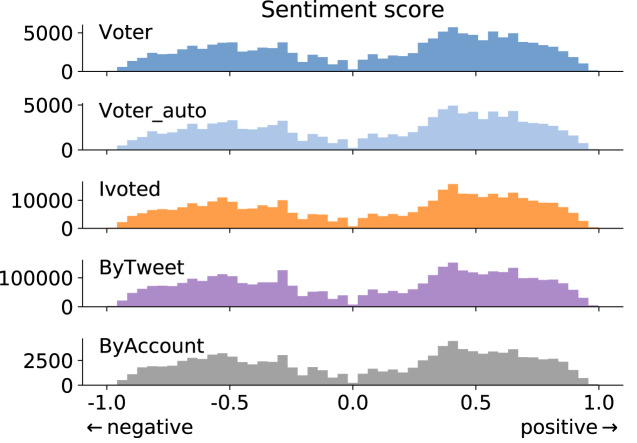
Tweet-level sentiment score distributions for different groups. Positive scores mean positive sentiment and *vice versa*. Sentiment-neutral tweets are excluded from this figure.

We show the tweet-level sentiment score distributions for different groups in Fig. 5. A score of zero is assigned to a tweet if it has no sentiment-expressing words. The percentage of neutral tweets is consistent across the groups (about 30%), so we excluded them from Fig. 5 to emphasize the patterns of positive and negative expressions. We find that the general sentiment is consistent across groups.

### Political alignment

Since we are interested in behaviors regarding the U.S. elections, it is important to estimate the political alignment of accounts. Following previous studies, we rely on the content produced by each account for this analysis. We apply two independent methods, one using hashtags and one using URLs (the links in the tweets) (Chen et al., 2021). In both methods, a political alignment score is assigned to each entity (hashtag or URL) in the tweets. The alignment score of each tweet is then obtained by averaging the associated entity scores, and the average score across an account’s tweets is used as the alignment score of that account. Next, we briefly introduce how each method assigns political alignment scores to the entities.

Hashtags are widely adopted by Twitter users to label topics and political alignment for their tweets. Therefore it is common to use hashtags to infer user alignment (Conover et al., 2011; Cota et al., 2019). Instead of manually labeling hashtags, we apply a recently developed approach to obtain continuous alignment scores in a semi-supervised fashion (Chen et al., 2021). We first deployed the *word2vec* algorithm (Mikolov et al., 2013a, 2013b) to infer vectorized representations of the hashtags based on their co-occurrence. Since the vectors encode the semantic relations between the entities, the axis between a pair of carefully selected hashtags can represent the political spectrum (An, Kwak & Ahn, 2018; Kozlowski, Taddy & Evans, 2019). The projected position of a hashtag on this axis reflects its alignment. In this study, we used #voteblue and #votered as the two anchors of the political alignment axis. We then calculated the relative positions of the remaining hashtags on the axis to produce alignment scores in [−1,1] (negative values mean left-leaning).

Twitter users also commonly use links to share news and information from other platforms. Many websites, especially news outlets, have known political alignment, making it possible to infer an account’s political score based on shared links. We adopt a list of 500 news sources with political alignment scores in [−1,1] (negative values mean left-leaning) from previous work (Bakshy, Messing & Adamic, 2015). When processing the tweets, we extract the URLs, expand the shortened ones, obtain the domains, and then assign them scores from the list. Entities and tweets with no score assigned are excluded in the evaluation.

**Figure 6:**
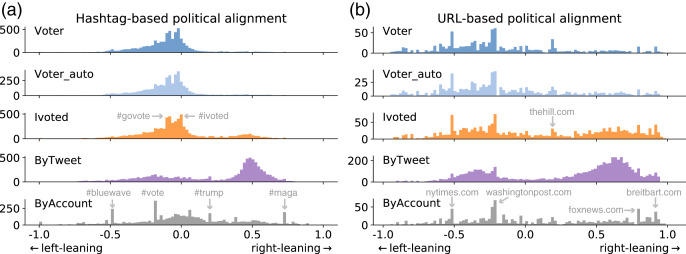
Distributions of political alignment scores for different groups using the (A) hashtag-based and (B) URL-based method. Peaks are due to popular entities, some of which are annotated.

We show the distributions of political alignment scores estimated using the two methods in Fig. 6. The two methods generate quantitatively different results but qualitatively similar patterns. They suggest that voter accounts tend to be left-leaning, in agreement with previous surveys (Mellon & Prosser, 2017; Wojcik & Hughes, 2019). Ivoted exhibits a similar distribution with Voter except that it has more right-leaning accounts. The ByAccount group presents a relative diverse and symmetrical distribution across the spectrum. Most surprisingly, accounts in the ByTweet group show a bimodal distribution with more accounts on the conservative side. This is inconsistent with the other groups, suggesting that the activity-weighted sampling bias completely distorts the political representation of Twitter accounts.

## Characterization of Questionable Behaviors

In this section, we focus on the questionable behaviors of the accounts in different sample groups. Specifically, we examine their automated activities, abnormal tweet deletions, sharing of low-credibility information, and account suspension statuses.

### Automated activities

As discussed in the introduction, various inauthentic accounts might participate in online discussions about elections. Therefore it is important to estimate the prevalence of these accounts in different groups. Since social bots are the only type whose prevalence can be statistically estimated using off-the-shelf detection tools, we focus on automated accounts in this subsection.

**Figure 7:**
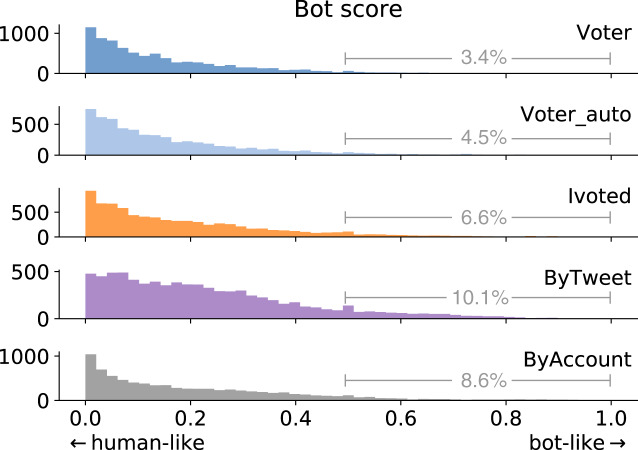
Bot score distributions of different account groups. We annotate the percentage of accounts having bot score above 0.5.

We adopted the bot detection model proposed by Yang et al. (2020). By strategically selecting a subset of the training dataset, the model achieves high accuracy in cross-validation as well as cross-domain tests. The classifier produces a bot score between zero and one for each account, where larger scores mean more bot-like. The bot score distributions for different groups are shown in Fig. 7. We can see that the Voter group has the lowest bot scores, while accounts in ByTweet tend to be more bot-like.

**Figure 8:**
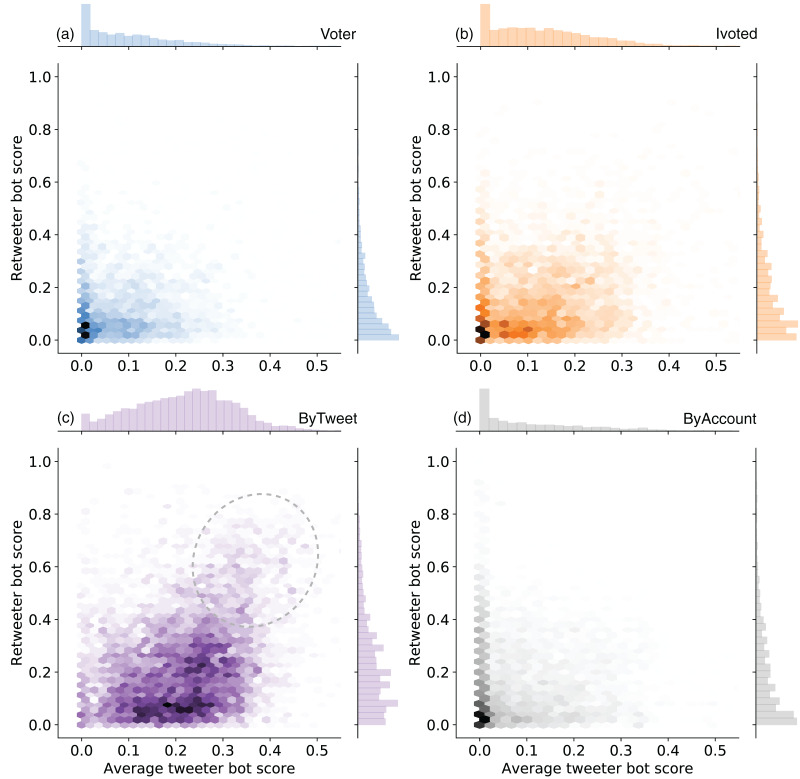
Joint distributions of the average tweeter bot scores and retweeter bot scores in (A) Voter, (B) Ivoted, (C) ByTweet, and (D) ByAccount groups. The plot for the Voter_auto group is identical to the Voter plot and omitted. Accounts without any retweets are ignored in this analysis.

Since the majority of political tweets in our dataset are retweets, we also checked whether the sampled accounts amplify messages from likely bots. We called the accounts being retweeted “tweeters” and the accounts amplifying the messages “retweeters.” For each retweeter account in the samples, we calculated the average tweeter bot scores weighted by the number of retweets. We plotted the joint distributions of average bot scores of tweeters and bot scores of retweeters in different groups in Fig. 8.

We see that the accounts amplified by the ByTweet group tend have higher average bot scores, indicating more bot-like behaviors. On the other hand, Voter, Voter_auto, and ByAccount groups seem to mainly engage human-like accounts. Ivoted lies in the middle. We also observe increased density in the top-right region (highlighted by an oval) in Fig. 8C, suggesting interactions among bot-like accounts in the ByTweet group.

### Abnormal tweet deletion

Twitter users have the right to remove their content and may do so for various legitimate reasons, from fixing typos to removing regrettable content (Almuhimedi et al., 2013; Zhou, Wang & Chen, 2016). However, this feature can be abused. For example, some politicians delete old tweets for public image management (Meeks, 2018). Trolls and bots may systematically delete their tweets to make their actions and intentions harder to detect (Zannettou et al., 2019a, Yang et al., 2019b).

Since we did not explicitly collect deletion events, we inferred them from our data. User objects that come with the tweets are snapshots of the account profiles at the time of tweet collection. A decrease in the total number of status counts between two consecutive tweets in the collection indicates a tweet deletion event. Note that not all deletion events can be detected with this method. We recorded the maximum drop in status counts for each account.

**Figure 9:**
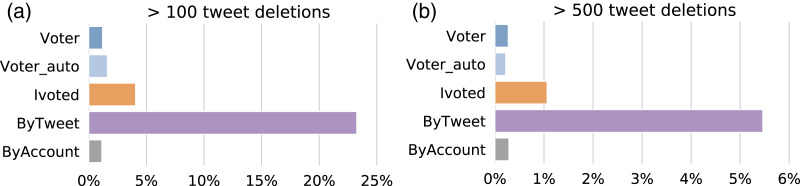
Percentages of accounts that delete more than (A) 100 and (B) 500 tweets at once in different groups.

To analyze abnormal tweet deletion behaviors, we first need to establish a reference deletion frequency. We adopted numbers from a previous study showing that Twitter users delete seven to 11 tweets on average in a week (Almuhimedi et al., 2013). In our case, the data collection continued for about three months, so we consider a status count drop of 100 or more tweets as a conservative threshold of abnormal behavior. Figure 9 shows the percentage of accounts with abnormal tweet deletion behaviors in each group. The ByTweet group has a much higher ratio of accounts that exhibit abnormal tweet deletion behaviors; this result is robust with respect to higher thresholds.

### Accounts suspension

**Figure 10:**
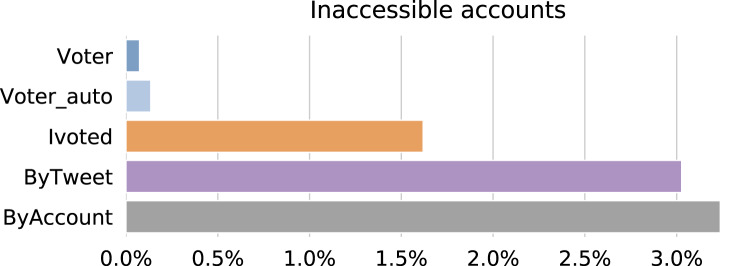
Percentages of accounts that were inaccessible as of 05 January, 2019 in different groups.

In the 2018 midterm election season, Twitter reinforced their actions against suspicious activities and suspended many accounts that violated their policy (https://blog.twitter.com/en_us/topics/company/2019/18_midterm_review.html). In line with previous studies (Alothali et al., 2018), we consider being suspended by the platform as a signal of illicit behaviors. We checked the status of the accounts on 05 January, 2019 through the Twitter user lookup API and plot the percentages of inaccessible accounts in different groups in Fig. 10. Being inaccessible means the target account is either suspended, deleted, or protected. Users can delete or protect their accounts, so inaccessible accounts are not necessarily suspicious. However, such actions are very rare compared to suspension, so we use inaccessibility as a proxy for suspension.

Interestingly, ByAccount has the highest suspension rate this time followed closely by ByTweet. This suggests that from Twitter’s policy standpoint, hyperactivity is neither a flag of illicit behavior nor correlated with illicit behaviors. Accounts in Ivoted also suffer from suspensions to some extent, while most accounts in Voter and Voter_auto are not affected.

### Sharing low-credibility information

Concern about low-credibility information spreading on social media has been growing ever since the 2016 U.S. presidential election. It is therefore interesting to evaluate the level of involvement for accounts in different groups. We focused on the URLs extracted from the collected tweets and evaluated the credibility of the content at the domain level following the literature (Shao et al., 2018; Grinberg et al., 2019; Guess, Nagler & Tucker, 2019; Pennycook & Rand, 2019; Bovet & Makse, 2019; Vosoughi, Roy & Aral, 2018). We compiled a list of low-credibility domains from recent research papers. A domain was labeled as low-credibility if it is either (a) labeled as low-credibility by Shao et al. (2018); (b) labeled as either “fakenews” or “hyperpartisan” by Pennycook & Rand (2019); (c) labeled as “fakenews,” “extremeright,” or “extremeleft” by Bovet & Makse (2019); or (d) labeled as “Black,” “Red,” or “Satire” by Grinberg et al. (2019). This procedure yielded 570 low-credibility domains. Note that we treat hyperpartisan domains as low-credibility.

We followed the same procedure as in the political alignment estimation to obtain the URL domains from each tweet. We excluded from the analysis URLs linking to Twitter itself and other social media like Facebook and YouTube. Most other domains were news outlets. With the help of the list, we are able to characterize the sharing of low-credibility information by the accounts.

**Figure 11:**
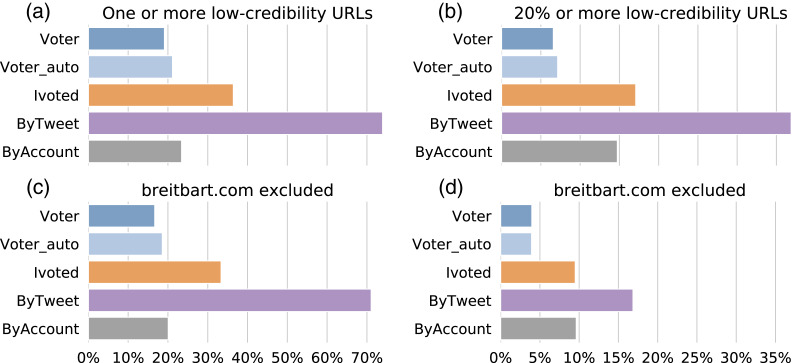
Sharing links to low-credibility sources. (A) Percentages of accounts in different groups sharing at least one URL from low-credibility sources. (B) Percentages of accounts in different groups having at least 20% of shared URLs from low-credibility sources. (C) Same as (A) but breitbart.com is excluded. (D) Same as (B) but breitbart.com is excluded.

First, we calculated the percentage of accounts that shared at least one low-credibility URL for each group and plot the results in Fig. 11A. We found that the number for ByTweet is alarmingly high—over 70% of the accounts had shared links to low-credibility sources. The other groups have lower percentages. We noticed that breitbart.com appears much more frequently than other low-credibility domains in our dataset. To make sure the pattern observed in Fig. 11A is not dominated by one hyperpartisan source, we performed a robustness test by excluding this domain and show the results in Fig. 11C. The proportions decrease slightly for all groups, but the results were unaffected.

The higher portion of links to low-credibility sources shared by the ByTweet group could be trivially due to the higher number of tweets produced by accounts in this group. To exclude this possibility, let us also consider the proportion of low-credibility URLs shared by each account. In Fig. 11B, we show the percentages of accounts with more than 20% of their shared links from low-credibility sources. The results were qualitatively similar, but the percentage of accounts reduces by about half. We also performed the same robustness check by excluding breitbart.com and plot the results in Fig. 11D. The percentages drop further, but the general pattern is similar.

**Figure 12:**
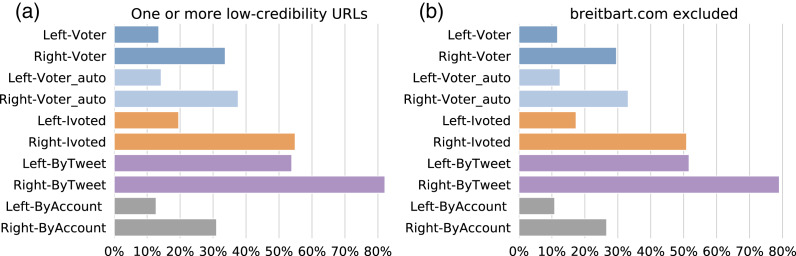
Sharing links to low-credibility sources, by political alignment. (A) Percentages of accounts in different sub-groups that shared at least one URL from low-credibility sources. (B) Same as (A) but breitbart.com is excluded.

In addition to treating each group as a whole, we can break them down by political alignment. We reported the results for left- and right-leaning accounts from each group in Fig. 12. By comparing accounts with the same political alignment across the groups, we find that the same patterns as before: ByTweet accounts have the highest rate of low-credibility content. Within each group, the right-leaning accounts were more likely to share links to low-credibility sources. We performed the same analysis using the proportion of low-credibility URLs (not shown), with identical observations.

## Discussion

To probe genuine opinions from just legitimate users, proper filtering of Twitter accounts is necessary. The ideal ground truth would be a representative sample of Twitter users who are also voters. We evaluate multiple methods that could be used to obtain such an ideal sample. This article introduces a novel method based on the *#ivoted* hashtag and corresponding pictures to find self-identified voters. Our analyses confirmed that manually-identified voters are least likely to display suspicious behaviors. We also evaluated the performance of two more efficient and lower-cost variants of this method. Using just the *#ivoted* hashtag (Deb et al., 2019) is simplest and cheapest, but results based on this sampling method are still subject to some bias along all the dimensions studied. The alternative automated approach is also efficient in that no human resources are required for manual annotation, at the cost of losing some precision and recall. Our systematic comparison reveals that this sampling method is as reliable as the manual method: the Voter_auto group is almost identical to Voter along all of the dimensions studied.

These methods have some limitations. First, unlike survey-based methods, our approaches cannot provide accurate demographic information about the voters. Second, only voters who are willing to reveal their voting actions online can be identified. This could introduce biases based on, say, age and privacy preferences. Third, the number of identified voters could be limited due to the strict criteria, for example, in cases when there are no hashtags widely adopted to self-identify voters. The resulting smaller size of the sample might negatively affect its statistical power. Finally, the proposed methods could be exploited by malicious actors, *e.g*., to target voter suppression efforts.

Nevertheless, we believe our approaches have their merit. The manual and automated voter identification methods are efficient and inexpensive compared to traditional ones. They can be applied to future elections in the U.S. and other countries with similar social norms around voting. The methods can be applied to other platforms like Instagram and Facebook where people share photos of themselves wearing “I Voted” stickers.

We further explore the biases in a naive account sampling method based on tweet volume. Compared with the identified voters, accounts in ByTweet are hyperactive: they tend to flood the platform with political content and aggressively expand their social networks while holding a right-leaning political viewpoint. When it comes to questionable behaviors, ByTweet accounts tend to show more bot activity and suspicious deletions as well as share more low-credibility information. These findings suggest that the Twitter stream is dominated by these hyperactive accounts, who introduce political bias and inauthentic signals. For researchers, the naive sampling approach could fundamentally bias their studies in the context of political elections. Normal Twitter users could also be disproportionately influenced *via* direct exposure (Shao et al., 2018), indirectly *via* social engagement metrics (Avram et al., 2020), or through platform mechanisms like trending topics and feed ranking algorithms, which use social engagement metrics as signals (Ciampaglia et al., 2018).

Our analyses of the ByAccount group suggest that randomly sampling accounts with equal weights could be another effective way to reduce bias and inauthentic signals. Accounts in the ByAccount group are similar to likely voters and only exhibit mild suspicious behaviors, although they do have a high suspension rate. For high quality data, one may consider applying bot and troll detection algorithms in the literature to identify and remove likely inauthentic accounts from such a sample (Sayyadiharikandeh et al., 2020; Addawood et al., 2019).

## Conclusion

In this article, we propose a method to identify voters on Twitter and systematically compare their behaviors with different samples of Twitter accounts in the context of U.S. midterm elections. We found that compared to likely voters and randomly selected accounts, accounts sampled from the Twitter stream tend to be hyperactive and markedly more conservative. They also exhibit various suspicious behaviors, are involved with more automated activities, and share more links to low-credibility sources. Researchers and journalists who analyze political issues using social media data must be mindful of these pitfalls of sampling biases.

## Additional Information and Declarations

### Data Availability

The following information was supplied regarding data availability: The data and code are available at Zenodo: Kai-Cheng Yang, Pik-Mai Hui, & Filippo Menczer. (2022). Dataset for paper: How Twitter Data Sampling Biases U.S. Voter Behavior Characterizations [Data set]. Zenodo. https://doi.org/10.5281/zenodo.6547792.
